# Role of molecular turnover in dynamic deformation of a three-dimensional cellular membrane

**DOI:** 10.1007/s10237-017-0920-8

**Published:** 2017-05-29

**Authors:** Satoru Okuda, Mototsugu Eiraku

**Affiliations:** 1 Laboratory for in vitro Histogenesis, Center for Developmental Biology (CDB), RIKEN, 2-2-3 Minatojima-minamimachi, Chuo-ku, Kobe, Hyogo 650-0047 Japan; 2 JST PRESTO, 4-1-8 Honcho, Kawaguchi, Saitama 332-0012 Japan

**Keywords:** Cytomembrane, Molecular turnover, Triangular mesh model, Mechanosensing, Coarse-grained modeling, Multiscale simulation

## Abstract

In cells, the molecular constituents of membranes are dynamically turned over by transportation from one membrane to another. This molecular turnover causes the membrane to shrink or expand by sensing the stress state within the cell, changing its morphology. At present, little is known as to how this turnover regulates the dynamic deformation of cellular membranes. In this study, we propose a new physical model by which molecular turnover is coupled with three-dimensional membrane deformation to explore mechanosensing roles of turnover in cellular membrane deformations. In particular, as an example of microscopic machinery, based on a coarse-graining description, we suppose that molecular turnover depends on the local membrane strain. Using the proposed model, we demonstrate computational simulations of a single vesicle. The results show that molecular turnover adaptively facilitates vesicle deformation, owing to its stress dependence; while the vesicle drastically expands in the case with low bending rigidity, it shrinks in that with high bending rigidity. Moreover, localized active tension on the membrane causes cellular migration by driving the directional transport of molecules within the cell. These results illustrate the use of the proposed model as well as the role of turnover in the dynamic deformations of cellular membranes.

**Electronic supplementary material:**

The online version of this article (doi:10.1007/s10237-017-0920-8) contains supplementary material, which is available to authorized users.

## Introduction

Cellular membranes dynamically extend and shrink by removing and replacing their molecular components such as the constituents of their phospholipid and lining cytoskeletons (Staykova et al. 2011). For example, during the lamellipodia formation, the cytomembrane projects outwards as lipid molecules diffuse from within the surrounding membrane and are transported from the cytoplasm (Keren 2011). Moreover, in the process of epithelial mitosis, cells deform to become round as they decrease their surface area, with lipid molecules being transported into the cytoplasm (Raucher and Sheetz 1990). In these cases, membrane deformations are actively driven by forces generated within the cell, i.e., actomyosin contractile forces. Importantly, in general, the membrane morphology is geometrically constrained by its volume–area balance (Ghosh and Singh 1992). Owing to this constraint, deforming cellular membranes requires changing the number of membrane molecules. Thus, molecular turnover has a crucial role in regulating cellular membrane deformations.

**Fig. 1:**
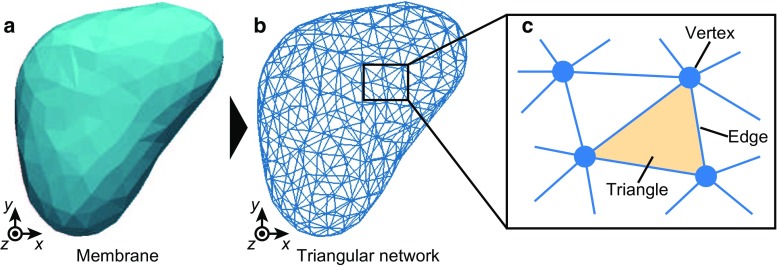
Triangular membrane model. **a** Single membrane. **b** Triangular network composing the single membrane shown in **a**. **c** Schematic diagram of the triangular network. Membrane morphology is expressed by a patch of *triangles*, wherein individual triangles share their vertices and edges with their neighbors

This turnover can be regulated by membrane-associated proteins (Peters et al. 2016; McMahon and Gallop 2005; Kozlov et al. 2010). On a molecular scale, these proteins are collectively localized on the membrane and form complexes to pinch off parts of the membrane as liposomes. These liposomes are transported to another membrane and fused through the activities of membrane-associated proteins, such as SNAREs (Grant and Donaldson 2009). Notably, these proteins are known to play the role of mechanosensors, owing to the dependence of adhesion upon the membrane-stress state (Kozlov et al. 2010). Therefore, understanding the effects of turnover upon membrane deformation requires analyzing the feedback from the membrane-stress state on the turnover.

Several computational methods have been proposed to analyze the dynamics of cellular membranes. At a molecular level, molecular dynamics methods have been often used, whereby individual atoms are expressed as particles. Because the membrane dynamics are realistically expressed on the scale of individual lipid molecules, turnover in these methods physically results from molecular interactions (van der Ploeg and Berendsen 1982; Heller et al. 1993; Chiu et al. 1995). On the contrary, at a continuum level, several coarse-graining models have been proposed, ignoring the degrees of freedom of individual lipid molecules (Gompper and Kroll 1997; Ho and Baumgärtner 1990; Boal and Rao 1992; Gompper and Kroll 2004; Zhao and Kindt 2005). In particular, triangular mesh models have often been used to analyze the macroscopic dynamics of organelles and cytomembranes (Noguchi and Gompper 2005b; Ramakrishnan et al. 2013, 2015), and also applied to the analyses of cellular mechanotransduction (Atilgan and Sun 2007; Powers et al. 2012, 2014). In these models, membrane morphology is expressed by a triangular meshwork and membrane fluidity is successfully expressed by dynamically remodeling the meshwork topology (Gompper and Kroll 1998). Thus, modeling the membrane turnover on a triangular mesh model yields a powerful tool for investigating the dynamics of cellular membranes.

In this study, we propose a new computational model for simulating the turnover-dependent dynamics of three-dimensional cellular membranes. Firstly, we propose topological operations on a triangular network to express the plastic extension and shrinkage of a membrane. Secondly, we propose stochastic descriptions of molecular transport that depend upon the membrane-stress state. Thirdly, using the proposed model, we demonstrate computational simulations of several membrane dynamics and explore the effects of turnover upon membrane deformation. Finally, we discuss the applicability of the proposed model and report new findings on the effects of turnover upon membrane deformations.

## Multiscale modeling of turnover-dependent membrane dynamics

### Description of three-dimensional membrane deformation

The membrane shape is expressed by a triangular meshwork (Fig. 1a), whereby the membrane surface is expressed by a patch of triangles (Fig. 1b, c).

The membrane dynamics are governed by an equation for the motion of vertices. By representing the position vector of the *i*th vertex by $\varvec{r}_{i}$, the vertex motion obeys the over-dumped Langevin equation as follows:

1 $$\begin{aligned} \eta \frac{\mathrm{d} \varvec{r}_i}{\mathrm{d} t} = - \nabla U + \varvec{w}_i. \end{aligned}$$

The left-hand side of Eq. (1) indicates a frictional force exerted on the *i*th vertex. Scalar $\eta$ is a friction coefficient between the membrane and its microenvironment. The right-hand side of Eq. (1) denotes the energetic force acting on the *i*th vertex, where *U* is an effective energy function. Variable $\varvec{w}_i$ is the Gaussian noise exerted on the *i*th vertex that satisfies the following statistics:

2 $$\begin{aligned}&\left\langle \varvec{w}_i \left( t \right) \right\rangle = \varvec{0}, \end{aligned}$$

3 $$\begin{aligned}&\left\langle \varvec{w}_i \left( t \right) \otimes \varvec{w}_j \left( t' \right) \right\rangle = 2 \eta k_B T \delta _{ij} \delta \left( t -t' \right) \varvec{1}, \end{aligned}$$

where $\left\langle ... \right\rangle$ is a statistical average, $\varvec{0}$ is the zero vector, and $\varvec{1}$ is the second-order unit tensor. Constant $k_B$ is the Boltzmann constant and *T* is the effective temperature.

Effective energy *U* is given by

4 $$\begin{aligned} U = U_\text {cc} + U_\text {eff} + U_\text {act}, \end{aligned}$$

where $U_\text {cc}$, $U_\text {eff}$, and $U_\text {act}$ are the constraint, effective, and active energy functions, respectively.

From a numerical viewpoint, to maintain a discrete size for the triangular mesh, we describe the constraint energy, $U_\text {cc}$, by the following function:

5 $$\begin{aligned} \begin{aligned} U_\text {cc}&= \sum ^\text {vertex}_{i} \sum ^\text {vertex}_{j (>i)} \frac{K_\text {r}}{2} \left( \frac{l_{ij}}{l_{\text {rep}}} - 1 \right) ^2 \delta _{\left[ l_{ij} < l_{\text {rep}} \right] }, \end{aligned} \end{aligned}$$

where $\delta _{\left[ \alpha \right] }$ is a binary function of the condition $\alpha$. Here, $K_\text {r}$ and $l_{\text {rep} }$ are the repulsive modulus and distance, respectively.

Membrane mechanics are described by the effective energy, $U_\text {eff}$. Here, we introduce the *i*th cell volume $v_i$, the *i*th triangle area $a_i$, the mean curvature around the *i*th vertex $M_i$ and the mean surface area around the *i*th vertex $A_i$. Using these variables, $U_\text {eff}$ is simply described by

6 $$\begin{aligned} \begin{aligned} U_\text {eff}&= \sum ^\text {vesicle}_{i} \frac{K_\text {v}}{2} \left( \frac{v_i}{v_{\text {eq} i}} - 1 \right) ^2 \\&\quad + \sum ^{\text {triangle}}_{i} \frac{K_\text {a}}{2} \left( \frac{a_i}{a_{\text {eq}}} - 1 \right) ^2 \\&\quad + \sum ^\text {vertex}_{i} 2 K_\text {c} \left( \frac{M_i^2}{A_i} \right) . \end{aligned} \end{aligned}$$

The first term indicates the volume elastic energy of individual vesicles, where $K_\text {v}$ and $v_{\text {eq} i}$ are the volume elasticity and the equilibrium volume of the *i*th vesicle, respectively. The second term indicates the surface elastic energy of the membrane exerted on individual triangle areas, where $K_\text {a}$ and $a_{\text {eq} }$ are membrane-surface elasticity and equilibrium area, respectively. The third term indicates the bending rigidity of the membrane, as exerted on individual vertices, where $K_\text {c}$ is a membrane bending rigidity (Julicher 1996; Tsubota 2014). Variable $M_i$ denotes the total mean curvature around the *i*th vertex: $M_i = \sum _{j(i)}^\text {edge} l_j \theta _j / 4$, where index *j*(*i*) is the *j*th edge surrounding the *i*th vertex. Variable $A_i$ is the surface area around the *i*th vertex: $A_i = \sum _{j(i)}^\text {triangle} a_j / 3$, where index *j*(*i*) is the *j*th triangle surrounding the *i*th vertex.

### Description of membrane fluidity and turnover

Cellular membrane has a fluidity, which causes viscous dissipation during membrane deformation. Moreover, membrane molecules are transported between the target vesicle and the reservoir comprising other vesicles within cell. In this model, we regard individual triangular elements as comprising a constant number of membrane molecules. Namely, in the model, the molecules composing the target vesicle is explicitly expressed as the triangular elements, whereas we implicitly express the molecules within the reservoir as a variable. Then, the fluidity can be expressed by a rearrangement of the triangular network (Gompper and Kroll 1998; Noguchi and Gompper 2004, 2005a). Moreover, the turnover can be regarded as a conversion between the triangular elements of the membrane and the number of molecules within the reservoir. Namely, the vesicle size increases when molecules are transferred to and decreases when transferred away from its surface. Therefore, in the model, the fluidity and turnover are expressed from two standpoints: topology and mechanics.

#### Spatiotemporal scales of our scope

To express membrane fluidity and turnover, we argue spatiotemporal scales of our scope.

From a coarse-graining viewpoint, the target vesicle is composed of the large number of triangular elements. Hence, by representing the current number of triangular elements composing the target vesicle by $N_\text {t}$, $N_\text {t}$ satisfies the following relationship:

7 $$\begin{aligned} \frac{1}{N_\text {t}} \simeq \Delta _N, \end{aligned}$$

where $\Delta _N$ is a positive finite value much smaller than unit. Hence, the spatial scale of vesicle is much larger than that of local triangular elements.

Based on the spatial scale, we consider the relationship of the scales of the numbers of molecules in the target vesicle, reservoir, and the triangular element. Here, the constant number of molecules in the individual triangular elements is represented by $m_\text {u}$, the total number of molecules composing the target vesicle by $M_\text {t}$, and the total number of molecules in the reservoir by $M_\text {r}$. Then, $M_\text {t}$ can be denoted by

8 $$\begin{aligned} M_\text {t} = N_\text {t} m_\text {u}. \end{aligned}$$

Because the membrane molecules are transported between the target vesicle and the reservoir, $M_\text {t}$ and $M_\text {r}$ are approximately on the same scale:

9 $$\begin{aligned} M_\text {t} \simeq M_\text {r}. \end{aligned}$$

Therefore, $M_\text {t}$, $M_\text {r}$, and $m_\text {u}$ satisfy the following relationship:

10 $$\begin{aligned} \frac{m_\text {u}}{M_\text {t}} \simeq \frac{m_\text {u}}{M_\text {r}} \simeq \Delta _N. \end{aligned}$$

By focusing on the local spatial scale of individual triangular elements, we consider the timescale of membrane fluidity and turnover, represented by $\tau _\text {f}$ and $\tau _\text {t}$, respectively. Here, $\tau _\text {f}$ is the characteristic time while the local state of individual triangular elements relaxes by the diffusion of membrane molecules, i.e., the viscous dissipation. $\tau _\text {t}$ is the characteristic time while the local state of individual triangular elements changes by the transportation of membrane molecules from/to the reservoir. Because the transportation of membrane molecules also requires their local diffusion, the time of local fluidity should be much faster than that of local turnover. Otherwise, the membrane should be locally broken during turnover. Therefore, $\tau _\text {f}$ and $\tau _\text {t}$ should satisfy the following relationship:

11 $$\begin{aligned} \frac{\tau _\text {f}}{\tau _\text {t}} \simeq \Delta _\tau , \end{aligned}$$

where $\Delta _\tau$ is a positive finit value much smaller than 1. This local relationship corresponds to the global cellular behaviors: While $\tau _\text {f}$ is the period over which the size of the whole cell seems approximately constant, as in blebbing, $\tau _\text {t}$ is the period over which the whole cell size dynamically varies, such as during proliferation or differentiation.

Notably, Eq. (11) indicates the timescales of the local dynamics within individual triangular elements, but not the global dynamics of the target vesicle. The timescales of the whole vesicle dynamics caused by the fluidity and turnover, represented by $\tau _\text {F}$ and $\tau _\text {T}$, can be estimated as

12 $$\begin{aligned} \tau _\text {F} := & {} M_\text {t} / \left( m_\text {t} / \tau _\text {f} \right) = N_\text {t} \tau _\text {f}, \end{aligned}$$

13 $$\begin{aligned} \tau _\text {T} := & {} M_\text {t} / \left( m_\text {t} / \tau _\text {t} \right) = N_\text {t} \tau _\text {t}, \end{aligned}$$

respectively. By comparing the amounts of $\Delta _N$ and $\Delta _\tau$, from Eqs. (8), (11), (12) and (13), the following relationship is given:

14 $$\begin{aligned} {\left\{ \begin{array}{ll} \tau _\text {T}> \tau _\text {t} \ge \tau _\text {F}> \tau _\text {f} &{} \text {when} \ \tau _\text {t} / \tau _\text {f} \ge N_\text {t}, \\ \tau _\text {T}> \tau _\text {F}> \tau _\text {t} > \tau _\text {f} &{} \text {when} \ \tau _\text {t} / \tau _\text {f} < N_\text {t}. \end{array}\right. } \end{aligned}$$

In case with $\tau _\text {t} / \tau _\text {f} \ge N_\text {t}$, effects of turnover on membrane are immediately relaxed within the whole vesicle. Hence, in the mesoscopic timescale $\tau _\text {T}> t > \tau _\text {f}$, the vesicle dynamics seem to be dominated by turnover through the number of membrane molecules. On the other hand, in case with $\tau _\text {t} / \tau _\text {f} < N_\text {t}$, effects of local turnover on membrane spend time to relax within the whole vesicle. Hence, in the mesoscopic timescale $\tau _\text {T}> t > \tau _\text {f}$, the vesicle dynamics seem to be affected by both turnover and fluidity. While the model covers the both cases, this study mainly focuses on the dynamics in case with $\tau _\text {t} / \tau _\text {f} < N_\text {t}$, by assuming large vesicles such as cytomembrane.

**Fig. 2:**
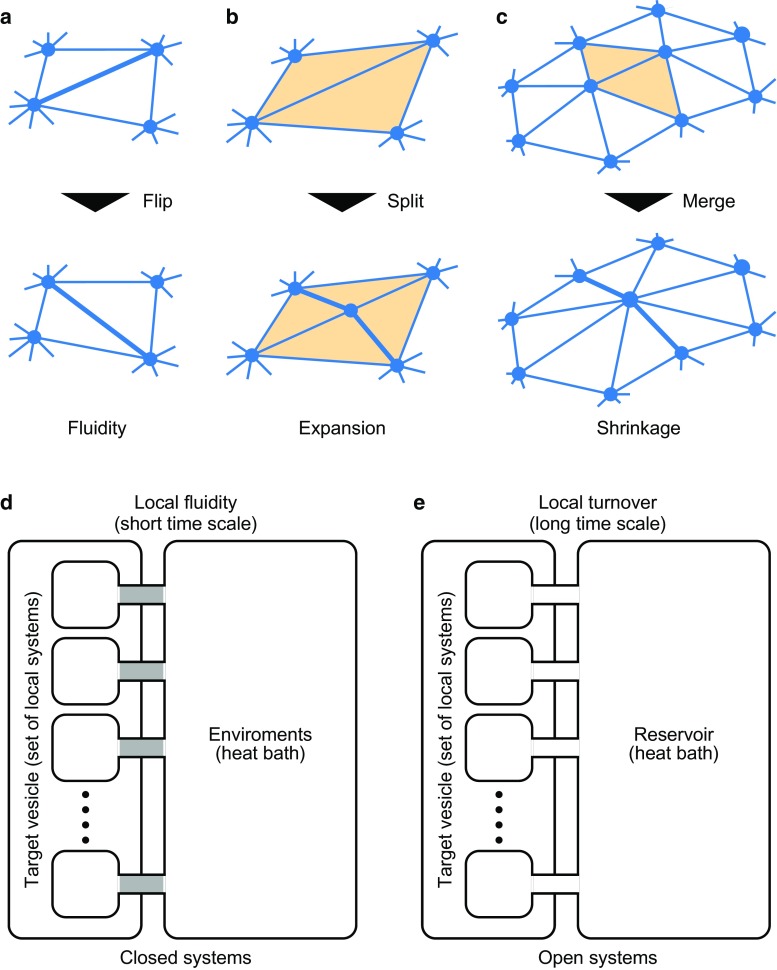
Topological and thermodynamic model of membrane fluidity and turnover. **a** Topological flipping operation for expressing membrane fluidity. **b** Topological splitting operation for expressing membrane expansion. Here, *two triangles* (*yellow triangles* in *top*) are split into *four triangles* (*yellow triangles* in *bottom*) along two edges (*thick lines* in the *bottom*). **c** Topological merging operation for expressing membrane shrinkage. Here *two triangles* (*yellow triangles* on *top*) are merged with *surrounding triangles* by being altered by the addition of two edges (*thick lines* in *bottom*). **d** Thermodynamic system of local membrane fluidity. **e** Thermodynamic system of local membrane turnover. In **d**, **e**, by regarding the target vesicle as a set of local systems, we consider the systems over a short timescale of fluidity and a long timescale of turnover, respectively

#### Expression of membrane fluidity

From a topological viewpoint, the membrane fluidity is modeled by flipping the edges of a couple of neighboring triangles (Gompper and Kroll 1998) (Fig. 2d). Moreover, from Eq. (11), the number of membrane molecules within individual vesicles can be approximately regarded as constant during viscous dissipation. Hence, according to a statistical mechanics, the local state around each edge can be regarded as obeying the canonical ensemble. Thus, the flip frequency of the *i*th edge, represented by $P_{\text {f} i}$, is given as the following probability:

15 $$\begin{aligned} P_{\text {f} i} = \frac{1}{\tau _\text {f}} \exp \left( - \frac{\Delta _i U}{k_BT} \right) , \end{aligned}$$

where variable $\Delta _i U$ indicates a gap in the total energy before and after flipping the *i*th edge. Notably, $\tau _\text {f}$ reflects the magnitude of membrane viscosity (Noguchi and Gompper 2004, 2005a).

#### Expression of membrane turnover

From a topological viewpoint, the turnover is modeled differently for the increase and decrease in the vesicle size. The increase in the vesicle size is expressed by the local network extension: splitting a couple of neighboring triangles (Fig. 2b). The decrease in the vesicle is expressed by the local network shrinkage: merging a couple of neighboring triangles with their surroundings (Fig. 2c). In these process, the position of a new vertex is determined as the center of the edge shared by the neighboring triangles.

Moreover, from Eq. (11), the local state around each edge, from a statistical-mechanical viewpoint, can be regarded as obeying a grand canonical ensemble. Here, we introduce the difference in effective energy before and after the split and merge around the *i*th edge, represented by $\Delta _i E$, as well as the chemical potential of the reservoir, represented by $\mu _\text {r}$. The frequency of the split and merge around the *i*th edge, represented by $P_{\text {t} i}$, is given by the following probability:

16 $$\begin{aligned} P_{\text {t} i} \propto \frac{1}{\tau _\text {t}} \exp \left( - \frac{ \Delta _i E \mp 2 m_\text {u} \mu _\text {r}}{k_BT} \right) , \end{aligned}$$

where the sign $\left( \mp \right)$ is negative for splitting and positive for merging. The factor of 2 multiplying the chemical potential originates from the number of triangles transformed by splitting and merging operations.

## Computational simulation of turnover-dependent membrane dynamics

### Introducing turnover behavior into the proposed model

In order to analyze effects of turnover on vesicle dynamics, we simply model the mechanosensing regulation using $\Delta _i E$ and $\mu _\text {r}$ in Eq. (16).

Because the local molecular transport depends on the local stress state of membrane, $\Delta _i E$ should be a function of vertex positions. As an example, we simply consider the dependency of turnover upon surface-area strain. Because the processes of vesicle fission and fusion require the activities of membrane-associated proteins, $\Delta _i E$ should involve an active energy cost in addition to passive energy difference such as the change in membrane curvature energy. Moreover, because the density of molecules composing the membrane is likely to be constant, turnover frequency seems approximately proportional to membrane-area strain. Hence, using the first-order approximation, we suppose a linear dependence of turnover upon membrane-area strain. Therefore, by introducing the average surface area of two split or merged triangles adjacent to the *i*th edge, represented by $\left\langle a_i \right\rangle$, we define $\Delta _i E$ as follows:

17 $$\begin{aligned} \Delta _i E = \epsilon _\text {t} \left\{ 1 \mp \frac{1}{\gamma _\text {t}} \left( \frac{\left\langle a_i \right\rangle }{a_{\text {eq}}} - 1 \right) \right\} , \end{aligned}$$

where the sign of $\left( \mp \right)$ is negative for splitting and positive for merging. Constant $\epsilon _\text {t}$ is the energetic cost of molecular turnover. The constant $\gamma _\text {t}$ is a critical strain for the energetic reduction of molecular turnover.

To simply define $\mu _\text {r}$, we consider an isolated system composed of the target vesicle and reservoir. Because membrane transport is actively driven by membrane-associated proteins, the active fluctuation of the turnover is much larger than the thermal fluctuation. Hence, by ignoring osmotic pressure, we focus on the active fluctuation around equilibrium.

For simplification, we assume that the probability density function of $M_\text {t}$ as the Gaussian distribution with a mean $M_\text {eq}$ and standard deviation $M_\text {inst}$ under the condition with $\Delta _i E = 0$. Here, $M_\text {inst}$ can be regarded as the instability of the number of molecules within the target vesicle. This assumption corresponds to defining $\mu _\text {r}$ as follows:

18 $$\begin{aligned} \mu _\text {r} = - \frac{\partial G_\text {r} }{\partial M_\text {t}}, G_\text {r} = \frac{k_BT}{ 12 m_\text {u} M_\text {inst}^2} \left| M_\text {t} - M_\text {eq} \right| ^3, \end{aligned}$$

where $G_\text {r}$ is the Gibbs free energy of the reservoir. Here, we introduce the current number of molecules within the vesicle, represented by $M_\text {t}$, as a continuum quantity. Then, to convert the triangular elements into molecules, we substitute Eq. (8).

By assuming the mass conservation law in the total number of molecules within cell, represented by $M_\text {tt} := M_\text {r} + M_\text {t}$, $M_\text {tt}$ is constant. Therefore, $\mu _\text {r}$ can be rewritten as follows:

19 $$\begin{aligned} \mu _\text {r} = \frac{\partial G_\text {r} }{\partial M_\text {r}}, G_\text {r} = \frac{k_BT}{ 12 m_\text {u} M_\text {inst}^2} \left| M_\text {r} - M_\text {req} \right| ^3, \end{aligned}$$

where $M_\text {req}$ is the equilibrium number of molecules in the reservoir: $M_\text {req} = M_\text {tt} - M_\text {eq}$. Namely, the employed assumption of $M_\text {t}$ corresponds to the second-order approximation of the active fluctuation of the number of molecules within the reservoir under equilibrium.

### Non-dimensionalization and parameter setting

To solve Eq. (1), parameter values are normalized to have unit length (*l*), unit time ($\tau$), unit number of molecules (*m*), and unit energy ($k_BT$). Here, *l*, $\tau$, and *m* are set as $l=\left( a_{\text {eq}} \right) ^\frac{1}{2}$, $\tau = 0.1 \eta a_{\text {eq}} / k_BT$, and $m=m_\text {u}$. Hereafter, physical parameters are described as dimensionless values. In case where a specific membrane is focused upon, the physical parameters employed in the simulations can be determined based on those measured by experiments. By assuming the system temperature to be 310 K, unit energy $k_BT$ becomes $4.3 \times 10^{-20}$ J. Based on this, the values of $K_\text {c}$ employed in this study correspond to $4.3 \times 10^{-20}$–$4.3 \times 10^{-19}$ J. These values have the similar range of those of the dimyristoylphosphatidylcholine (DMPC), the plant thylakoid lipid digalactosyldiacylglycerol (DGDG), and other general lipid membranes (Duwe et al. 1990; Engelhardt et al. 1985; Schneider et al. 1984; Mutz and Helfrich 1990; Evans and Rawicz 1990; Kummrow and Helfrich 1991).

To establish that the proposed model successfully recapitulates turnover-dependent membrane dynamics, several parameters are varied, such as $K_\text {c}$ and $\tau _\text {t}$. The state under the initial condition is set as a single vesicle composed of 1000 triangles, which are equilibrated under the condition $K_\text {c} = 10$. The equilibrium volume of the vesicle is set to $v_\text {eq} = 2527$, which corresponds to the volume of the vesicle with the area $1000 a_\text {eq}$ and sphericity 0.85. The setting of the geometric constraint $l_{\text {rep}}$ in Eq. (5) is described in Appendix A. Moreover, to set physical parameters, the force balance among individual energy terms in Eqs. (5) and (6) is taken into account, as described in Appendix B. Numerical implementation and calculation is described in Appendix C. All model parameters are shown in Table 1.

### Proposed model successfully recapitulates turnover-dependent membrane dynamics

**Fig. 3:**
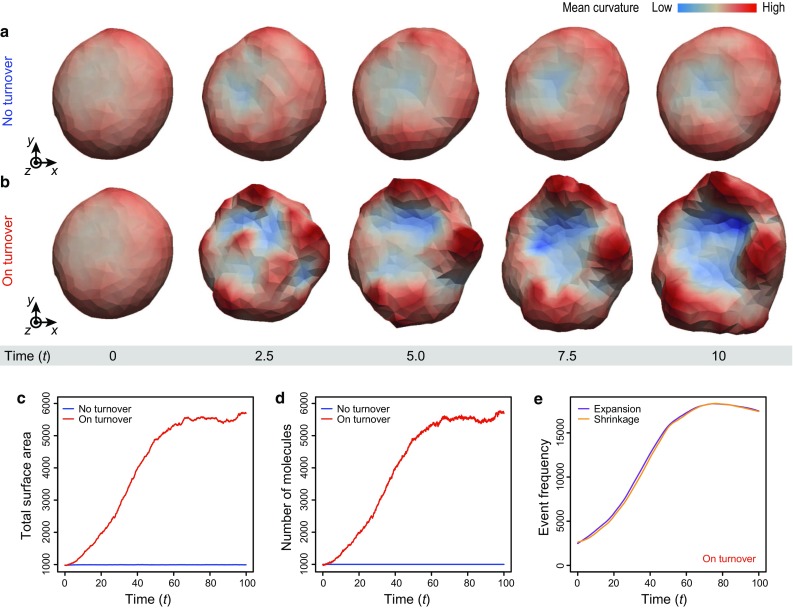
Turnover-dependent dynamics of cellular membranes. **a** Deformation process of the vesicle in the case without turnover ($\tau _\text {t} = +\infty$). **b** Deformation process of the vesicle in the case with turnover ($\tau _\text {t} = 1.0$). Vesicles are *colored* according to their local mean curvature. The dynamic process of **b** is also shown in Supplementary Movie 1. **c** Total surface area of the vesicle as a function of time *t*. **d** The number of membrane molecules as a function of time *t*. **e** Frequencies of expansion and shrinkage as functions of time *t*. The dynamics of **a** are calculated under condition $K_\text {c} = 3.0$. The dynamics of **b** are calculated under conditions $K_\text {c} = 3.0$ and $M_\text {inst} = 1000$

To establish whether the proposed model successfully recapitulates turnover-dependent membrane dynamics, we simulate vesicle dynamics in case with and without membrane turnover ($\tau _\text {t} = 1.0$ and $+\infty$) (Fig. 3). In the case without membrane turnover, the vesicle is slightly deformed by fluctuations while maintaining its surface area (Fig. 3a, c). On the other hand, in the case with turnover, the vesicle is significantly deformed as its surface expands (Fig. 3b, c). Hence, the large deformation is permitted by the surface-area extension (Fig. 3c). Moreover, for a long timescale, the total surface area and number of molecules reached the plateau (Fig. 3c, d). This tendency seems independent on the values of the bending rigidity $K_\text {c}$ and transport instability $M_\text {inst}$; meanwhile, we could not observe the plateau in the time range of our simulations in case with large $M_\text {inst}$. Importantly, the extension is caused by the increase in the number of molecules within the vesicle (Fig. 3d), but not by elastic deformation. In the process of this expansion, the number of molecules within the vesicle gradually increases in a stochastic manner (Fig. 3e). These results suggested that the proposed model successfully recapitulates the turnover-dependent membrane dynamics.

### Molecular turnover adaptively facilitates vesicle deformation

**Fig. 4:**
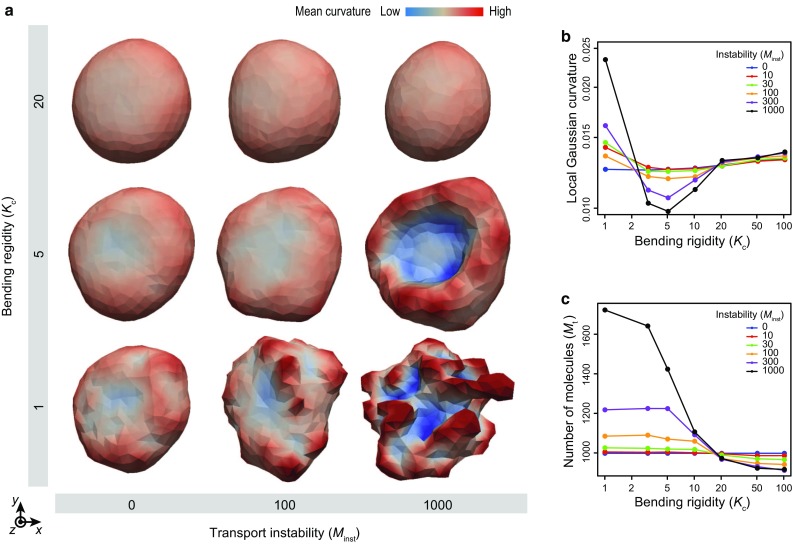
Effect of membrane turnover on membrane deformation. **a** Phase diagram of vesicle morphology for bending rigidity $K_\text {c}$ and transport instability $M_\text {inst}$ at $t = 10$. Vesicles are *colored* based on the local mean curvature. **b** Averaged local Gaussian curvature as a function of bending rigidity $K_\text {c}$ with respect to transport instability $M_\text {inst}$ at $t = 10$. **c** The number of molecules as a function of bending rigidity $K_\text {c}$, with respect to transport instability $M_\text {inst}$ at $t = 10$. These dynamics are calculated under the condition of $\tau _\text {t} = 1.0$

Next, to investigate the effects of molecular turnover upon membrane deformation, we analyze the effects of the bending rigidity $K_\text {c}$ and the instability modulus of the number of molecules within vesicle $M_\text {inst}$ (Fig. 4). To focus on the fundamental effects of turnover on membrane dynamics, we set $U_\text {act} = 0$ in Eq. (4) by assuming a simple membrane behavior.

The vesicle dynamics are found to drastically vary with respect to $K_\text {c}$ and $M_\text {inst}$ (Fig. 4a, b). In the case with small $M_\text {inst}$, the vesicle slightly deforms while maintaining its surface area. On the other hand, in case with large $M_\text {inst}$, the vesicle morphology drastically varies as it extends or shrinks with respect to $K_\text {c}$. In case with small $K_\text {c}$, the vesicle deforms to be lobate as its surface area increases. In case with large $K_\text {c}$, the vesicle deforms to be spherical as its surface area decreases.

**Table 1:** Model parameters

Symbol	Value	Description
*Parameters for physical behaviors*		
$K_\text {v}$	$1.0 \times 10^4$	Volume elasticity of vesicle
$K_\text {a}$	50	Surface elasticity of membrane
$K_\text {c}$	1.0–10	Bending rigidity of membrane
$\kappa _\text {act}$	1.0–3.0	Active surface energy on membrane
$v_\text {eq}$	2527	Equilibrium volume of vesicle
$\tau _\text {f}$	$1.0 \times 10^{-2}$	Characteristic time of membrane fluidity
$\tau _\text {t}$	1.0, $+\infty$	Characteristic time of membrane turnover
$M_\text {eq}$	$1.0 \times 10^3$	Equilibrium number of molecules within vesicle
$M_\text {inst}$	10–$1.0 \times 10^3$	Instability of the number of molecules within vesicle
$\epsilon _\text {t}$	$1.0 \times 10^{-1}$	Energetic cost of molecular turnover
$\gamma _\text {t}$	$5.0 \times 10^{-2}$	Critical strain for energetic reduction of molecular turnover
*Parameters for computational calculation*		
$K_\text {r}$	$1.0 \times 10^3$	Repulsive modulus
$\Delta t_\text {M}$	$1.0 \times 10^{-3}$	Time step of numerical integration

To analyze effects of $K_\text {c}$ on membrane shape, we measured the averaged local Gaussian curvature over every vertex, which is estimated from a set of the surrounding vertices. Notably, the averaged local Gaussian curvature cannot be conserved in the defiance of the Gauss–Bonnet theorem but dynamically vary. This is because the curvature is locally defined at individual vertices, whose number dynamically varies by turnover. Interestingly, the dependence of the averaged local Gaussian curvature on $M_\text {inst}$ changes directions at three areas; positive in cases $K_\text {c} \lesssim 2$, negative in cases $2 \lesssim K_\text {c} \lesssim 20$ and positive $20 \lesssim K_\text {c}$ (Fig. 4b). On the other hand, the number of membrane molecules is inversely proportional to $K_\text {c}$ independent on $M_\text {inst}$. These behaviors can be simply explained by the geometric constraint imposed by the volume–area balance; the surface must be finely folded in cases with large area ($K_\text {c} \lesssim 2$), laminarly flatted in cases with middle area ($2 \lesssim K_\text {c} \lesssim 20$) and smoothly spherical in cases with small area ($20 \lesssim K_\text {c}$). Therefore, the resulting membrane morphologies are regulated by the number of membrane molecules through turnover.

The turnover is dependent on $K_\text {c}$ because it reduces the local residual stress generated by the global force balance: in the case with low $K_\text {c}$, the membrane-surface area tends to expand because of the thermal fluctuation force. As $K_\text {c}$ increases, the membrane-surface area tends to decrease to minimize its bending energy. Therefore, the strain of the membrane-surface area $\left\langle a_i \right\rangle / a_{\text {eq}}$ is inversely proportional to the bending rigidity, $K_\text {c}$. By sensing local stress as Eq. (17), the molecular turnover is biased to cause expansion or shrinkage. These results suggest that the turnover serves to adaptively facilitate membrane deformation depending upon the membrane-stress state.

### Molecular turnover permits autonomous cell migration

**Fig. 5:**
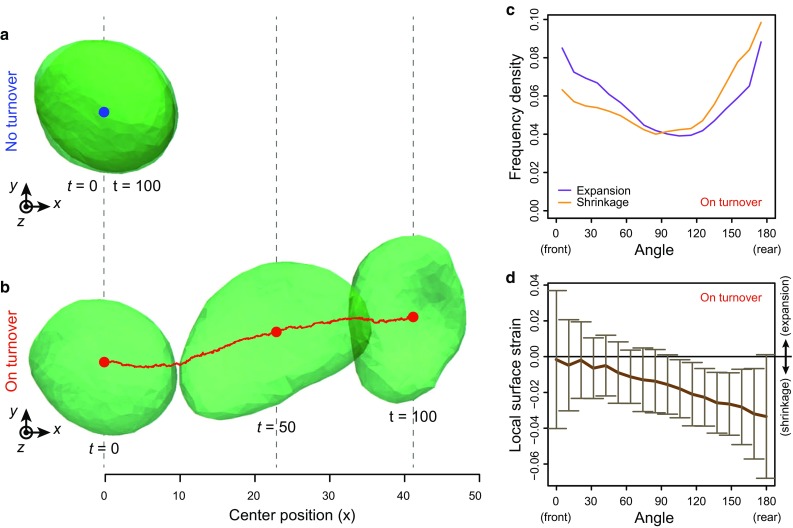
Effect of localized active tension on the membrane dynamics throughout turnover. **a** Vesicle dynamics without turnover ($\tau _\text {t} = +\infty$). **b** Vesicle dynamics with turnover ($\tau _\text {t} = 1.0$). The dynamic process of **b** is also shown in Supplementary Movie 2. **c** Normalized frequency densities of expansion and shrinkage in case with turnover as a function of the angle to the event site. **d** Local strain of the membrane surface in case with turnover as a function of the angle. The angle in **c**, **d** is defined as that between the *x*-axis and the vector from the center of the vesicle to the event site. In **c**, the frequency density is calculated as the local frequency per the surface area of sphere within the scope angle. In **d**, the local surface strain is calculated as the strain of individual triangular elements, and the *solid line* and *bar* indicate the average and standard deviation within the scope angle, respectively. These dynamics are calculated under the condition $K_\text {c} = 10$, $M_\text {inst} = 10$, and $K_\text {act} = 3.0$

Finally, to demonstrate the use of the model, we simulated cellular dynamics driven by an intracellular active force of lining cytoskeleton on membrane. To exert this force, we introduce a locally biased cortical surface energy as follows.

20 $$\begin{aligned} \begin{aligned} U_\text {act}&= \sum ^{\text {triangle}}_{i} \frac{ \kappa _\text {act} \left( 1 +\cos \phi _i \right) }{2} a_i \end{aligned} \end{aligned}$$

where $\phi _i$ is the constant defined at each time step as the angle between the *x*-axis and the vector from the center of the vesicle to the center of the *i*th triangle. This function is similar to that used in expressing the active energy on cells during collective migration (Sato et al. 2015).

As a result, in case without turnover, the velocity of migration drastically decreases (Fig. 5a, c). On the other hand, in case with turnover, the cell dynamically migrates along the *x*-axis (Fig. 5b, c). This is because the active energy in Eq. (20) drives the transport of molecules from the rear to the front through the turnover (Fig. 5d). This result suggests that cells can migrate even without any traction force on membrane, with turnover playing a crucial role in driving migration. Notably, while the migration can be observed in the wide range of $K_\text {c}$ and $M_\text {inst}$, $K_\text {c}$ must be high to maintain the spherical cell shape as described in Appendix E.

Notably, because the force from the energy in Eq. (20) is internal within cell but not external, the force is balanced within cell at each time. On the other hand, because $\phi _i$ dynamically varies with time under membrane deformation, *U* becomes non-conservative so as to set the system into non-equilibrium. Such non-conservative energy function has been known as to generate active cell movements (Sato et al. 2015). Moreover, the stress-dependency of the turnover in Eq. (16) breaks the detail balance of molecular transport. Thus, despite the force balance within cell, this model can generate cell migration in a physically consistent manner.

In biological systems, there are a lot of types of cell migration such as single and collective cell migration in wound healing, morphogenesis and cancer invasion (Friedl and Wolf 2003). Importantly, while mechanism of the resulting process is a kind of Brownian ratchet, it differs from the mechanism of the well-known single-cell migration. In this mechanism, the front extension of the cell initiates migration: It extends ahead by cytoskeletal polymerization and the rear follows it by cytoskeletal contraction. On the contrary, in our model, localized active tension at the rear initiates migration by directionally transporting molecules to the front. Notably, our model does not conflict with the mechanism of the single-cell migration. Therefore, turnover may contribute to cellular migration as a main or secondary process.

## Discussion

In this study, by making several physical assumptions, we integrally formulated the fluidity and turnover of a cellular membrane in a physically consistent manner. In the computational simulations, the cellular vesicles actively deform by the turnover of the membrane molecules (Fig. 3). During deformations, the triangular property was maintained to satisfy the modeling assumptions as in Appendix D. Moreover, based on the dependence of turnover upon the stress state, vesicle morphology drastically differed with the bending rigidity (Fig. 4); a smooth sphere was obtained under high rigidity, and a lobate vesicle was obtained under low rigidity. Furthermore, based on directional molecular transport by turnover, the localized active tension on the membrane drove the cellular migration of the vesicle (Fig. 5). From these results, the proposed model successfully recapitulated the turnover-dependent dynamics of three-dimensional cellular membranes.

In the computational simulations, the turnover behavior described in Eq. (16) was simply provided under the following assumptions: (i) that the energetic cost for molecular transport, $\Delta _i E$, depended on the local strain of the membrane area, as shown in Eq. (17) and (ii) that the chemical potential $\mu _\text {t}$ stabilized the number of molecules in the reservoir, as shown in Eq. (18). In general, these assumptions were not necessary for the proposed model. We emphasize that the functions of $\Delta _i E$ and $\mu _\text {r}$ can be entirely arbitrary.

Although the cellular mechanical property was expressed simply in these simulations, as described in Eq. (6), it can be expressed in more detail. For example, in cells with rich lining cytoskeletons, cell membranes could have non-negligible longitudinal and transverse elasticity, which may be important for their deformations (Heinrich et al. 2001). Moreover, during blebbing, the actin cytoskeleton lining membrane is locally broken, which causes a spatiotemporal inhomogeneity in membrane rigidity (Manakova et al. 2016). These details can be reflected by choosing a proper effective energy function, $U_\text {eff}$ in Eq. (6).

In principle, detailed expressions for turnover at a molecular scale are limited in the proposed model; for example, the adhesion process of membrane-associated proteins cannot be directly expressed. Such behaviors at the molecular scale must be instead coarse-grained into those at the minimum scale of the proposed model. For example, the dependence of protein adhesion upon lipid behavior can be expressed by $\Delta _i E$ as a dependence of the turnover upon membrane strain. Moreover, the biased adhesion of proteins to lipids can be expressed by $\mu _\text {r}$ as directional molecular transport. Thus, by designing $\Delta _i E$ and $\mu _\text {r}$, the proposed model can be applied to various behaviors of turnover.

The proposed model will help researchers understand the mechanics of 3D cellular dynamics involving membrane turnover, particularly the causal relationships between single-cell dynamics and the underlying molecular transport. The proposed model is a powerful approach for addressing the manner in which molecular turnover affects 3D cellular dynamics at a subcellular scale. Understanding these functions of turnover is necessary for better understanding cellular dynamics, and this will be useful as fundamental knowledge for controlling these dynamics in bioengineering. Moreover, by combining the proposed model at a subcellular scale with the models at a multicellular scale (Okuda et al. 2013, 2015), it will be possible to predict comprehensive cellular dynamics from molecules to tissues. Therefore, the proposed model will contribute to exploring the exploration of the frontiers of cellular biomechanics.

**Fig. 6:**
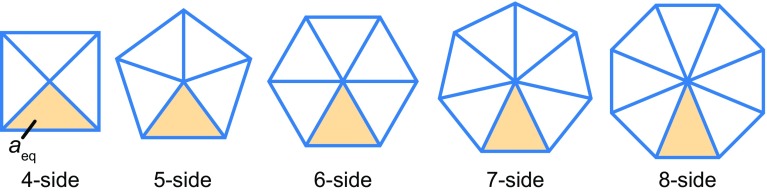
Regular in-plane *polygons* with *triangles* of the same area, $a_{\text {eq}}$, for determining the repulsive distance, $l_\text {rep}$

## Conclusion

To analyze the effect of molecular turnover on three-dimensional deformations of cellular membranes, we proposed a new computational model for simulating the turnover-dependent dynamics of three-dimensional cellular membranes. The proposed model successfully simulate turnover-dependent membrane dynamics, and suggested the roles of turnover to drive the adaptive deformation and directional migration of vesicles. These results illustrate the importance of turnover in the dynamic deformations of cellular membranes in addition to the use of the proposed model for exploring general effects of molecular turnover on cellular dynamics.

### Electronic supplementary material

Below is the link to the electronic supplementary material.

Supplementary material 1 (mp4 3148 KB)

Supplementary material 2 (mp4 6125 KB)

## Figures and Tables

**Fig. 7:**
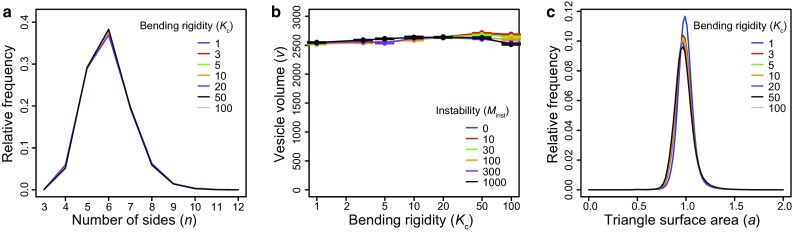
Membrane and triangular property. **a** Probability distribution of the number of triangular sides, *n*. **b** Vesicle volume, *v*, as a function of bending rigidity, $K_\text {c}$. **c** Probability distribution of *triangular area*, *a*. These probability distributions are calculated from the dynamics within the range of $9 \le t \le 10$. The dynamics in **a**, **c** are calculated under the conditions $\tau _\text {t} = 1.0$ and $M_\text {inst} = 1000$. The dynamics in **b** are calculated under the condition $\tau _\text {t} = 1.0$

**Fig. 8:**
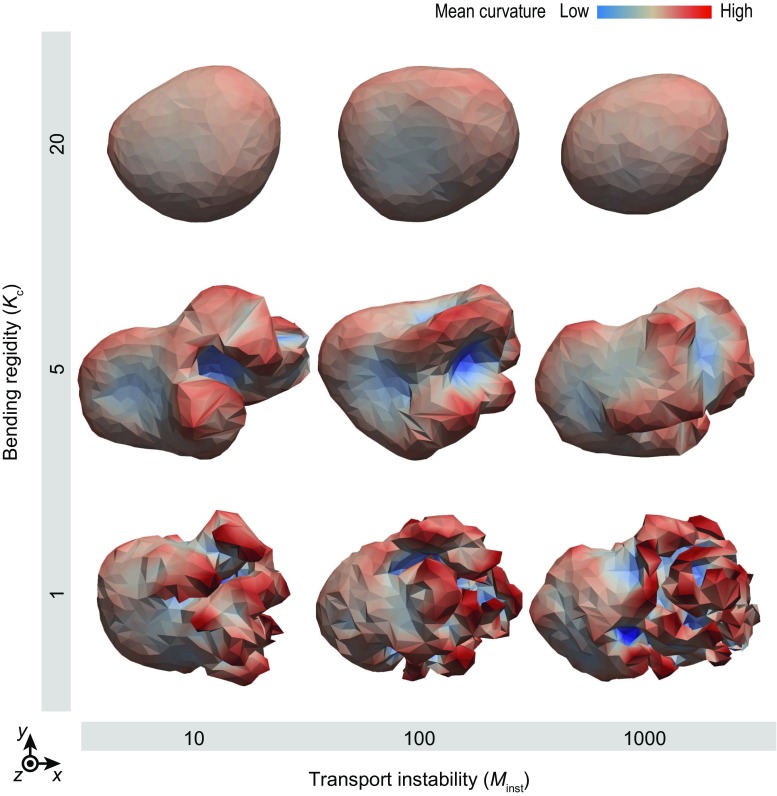
Effect of membrane turnover on cell morphology during migration. Cell morphologies at $t = 5$ are shown with respect to the bending rigidity $K_\text {c}$ and transport instability $M_\text {inst}$. Vesicles are *colored* based on the local mean curvature. These dynamics are calculated under the condition of $\tau _\text {t} = 1.0$ and $K_\text {act} = 1.0$

## Setting geometric constraint

Because the constraint energy $U_\text {cc}$ limits the distances between vertices, it can reduce the topological flexibility of the triangular meshwork. To determine the value of $l_\text {rep}$, we consider a *n*-side polygon surrounding a central vertex, wherein individual triangles have the same area $a_{\text {eq}}$. Then, the minimum lengths of edges of individual triangles can be given as a function of *n*. Then, to permit topological flexibility between 4- and 8-sided polygons (Fig. 6), we set $l_\text {rep}$ to be the minimum length of all of their edges as follows:

21 $$\begin{aligned} l_\text {rep} = 2 \left( \frac{a_\text {eq}}{\tan {\left( 7 \pi / 16\right) }} \right) ^{\frac{1}{2}}. \end{aligned}$$

Notably, because triangles can deform out-of-plane, the constraint condition does not rigidly limit the number of sides.

## Energetic force balance

From a physical viewpoint, the magnitudes of several parameters in Eq. (4) are related. First, because cellular membranes cannot cross over each other, the repulsive force in Eq. (5) should be enough large to act as a constraint. Second, because the cytoplasm and lipid membrane generally behave as incompressible liquids, the volume elasticity of the vesicles and the area elasticity of the membranes should be much larger than the other physical parameters. Moreover, because cellular membranes are constructed mainly from lipids, the bending rigidity of the membrane in Eq. (6) should be much smaller than the area elasticity of membrane. Therefore, individual forces exerted on vertices, represented by $- \nabla U$, should satisfy the following force balance:

22 $$\begin{aligned} \left| \nabla U_\text {cc} \right| \gg \left| \nabla U_\text {v} \right| \simeq \left| \nabla U_\text {a} \right| \gg \left| \nabla U_\text {c} \right| , \end{aligned}$$

where $U_\text {v}$, $U_\text {a}$, and $U_\text {c}$ are the first, second, and third terms of $U_\text {eff}$, respectively. Several constant parameters in Eq. (4) are set to satisfy Eq. (22).

## Numerical implementation and calculation

Monte Carlo simulations for sampling in Eqs. (15) and (16) were numerically conducted using the heat-bath algorithm at each interval of $\Delta t_\text {M}$. Time integration of Eq. (1) was numerically performed using the Euler method with a variable time step of $\Delta t_\text {E} ({\le }{\Delta t_\text {M}})$, which is dynamically determined to suppress the maximum vertex displacement. Because the frequencies of flip, split, and merge are enough low, the system state statistically reaches equilibrium during each interval of $\Delta t_\text {M}$. Because the error in the numerical integration ($O(\Delta t)$) is much smaller than the magnitude of thermal noise ($O(\Delta t^\frac{1}{2})$), we employed the first-order Euler method. All experiments were performed on a cluster computer comprising 12 nodes with 2.9 GHz Intel Xeon dual processors and 64 GB RAM.

## Validation of assumptions

In the proposed model, we made several assumptions: (i) that the timescales of membrane fluidity and turnover are related as in Eq. (11); (ii) that there is topological flexibility for membrane fluidity, and (iii) that the force balance is given by Eq. (22). Preassumption (i) is satisfied because it is given by the set of parameters. On the other hand, preassumptions (ii) and (iii) are non-trivial because they result from the dynamic process of membrane deformation. To ascertain whether these preassumptions are satisfied in the results, we analyze properties of the membrane and triangles. Firstly, to test the membrane fluidity, we analyze the probability distribution of the number of sides, which is broad from 4 to 9 (Fig. 7a). Hence, in the case where $l_\text {rep}$ is set as Eq. (21), the constraint energy permits the topological flexibility of the meshwork. Secondly, to test the balance of the individual energetic forces, we analyze the probability distributions of vesicle volume and triangular area. These distributions approximately equal those determined by the first and second terms of Eq. (6) (Fig. 7b, c), respectively. Hence, the preassumed force balance of Eq. (22) is satisfied. Moreover, from the distribution of triangular area in Fig. 7c, the discrete size of the triangular meshwork is approximately maintained. These results warrant certain predictions of the model from a physical viewpoint.

## Effect of membrane turnover on cell morphology during migration

To analyze the effect of molecular turnover on migration, we simulated cellular dynamics driven by the intracellular active force, described by Eq. (20). Figure 8 shows the cell morphologies during migration with respect to $K_\text {c}$ and $M_\text {inst}$. The results suggested that $K_\text {c}$ must be high to maintain the cell shape to be spherical. Otherwise, the cell surface strongly undulated.
